# Large Liver Blood Vessels and Bile Ducts Are Not Damaged by Electrochemotherapy with Bleomycin in Pigs

**DOI:** 10.1038/s41598-019-40395-y

**Published:** 2019-03-06

**Authors:** Jan Zmuc, Gorana Gasljevic, Gregor Sersa, Ibrahim Edhemovic, Nina Boc, Alenka Seliskar, Tanja Plavec, Maja Brloznik, Nina Milevoj, Erik Brecelj, Bor Kos, Jani Izlakar, Tomaz Jarm, Marko Snoj, Marina Stukelj, Damijan Miklavcic, Maja Cemazar

**Affiliations:** 1 Institute of Oncology Ljubljana, Zaloska cesta 2, 1000 Ljubljana, Slovenia; 2 University of Ljubljana, Veterinary Faculty, Gerbiceva ulica 60, 1000 Ljubljana, Slovenia; 3 University of Ljubljana, Faculty of Electrical Engineering, Trzaska cesta 25, 1000 Ljubljana, Slovenia

## Abstract

The first clinical studies on the use of electrochemotherapy to treat liver tumours that were not amenable to surgery or thermal ablation techniques have recently been published. However, there is still a lack of data on the effects of electrochemotherapy on normal liver tissue. Therefore, we designed a translational animal model study to test whether electrochemotherapy with bleomycin causes clinically significant damage to normal liver tissue, with emphasis on large blood vessels and bile ducts. We performed electrochemotherapy with bleomycin or delivered electric pulses alone using a potentially risky treatment strategy in eight pigs. Two and seven days after treatment, livers were explanted, and histological analysis was performed. Blood samples were collected before treatment and again before euthanasia to evaluate blood biomarkers of liver function and systemic inflammatory response. We found no thrombosis or other clinically significant damage to large blood vessels and bile ducts in the liver. No clinical or laboratory findings suggested impaired liver function or systemic inflammatory response. Electrochemotherapy with bleomycin does not cause clinically significant damage to normal liver tissue. Our study provides further evidence that electrochemotherapy with bleomycin is safe for treatment of patients with tumours near large blood vessels in the liver.

## Introduction

Electrochemotherapy (ECT) is a tumour treatment method that combines the use of electric pulses with cytotoxic drugs^1^. The exposure of tumour cells to sufficiently high electric fields facilitates transmembrane transport of otherwise poorly permeant cytotoxic drugs, such as bleomycin and cisplatin, due to reversible cell membrane electroporation^1^. The cytotoxicity of drugs is therefore increased up to several thousand times^2,3^. In addition to the increased uptake of cytotoxic drugs into cells, ECT also affects tumour blood flow through vasoconstriction (vascular lock mechanism)^4^ and the apoptosis of endothelial cells in tumour blood vessels (vascular disruption mechanism)^5,6^, as well as activating the immune response of the organism^7^. The nonthermal mechanism of action makes ECT an attractive treatment option for tumours in sensitive anatomical locations. Until now, ECT has mostly been used for the treatment of skin and subcutaneous tumours, for which standard operating procedures have been published^8^ and recently updated^9^. In a systematic meta-analysis of treatment effectiveness, single-session ECT of skin tumours resulted in a complete response in 59.4% and an objective response in 84.1% of cases^10^.

Recently, the use of ECT has expanded to deep-seated visceral tumours^11^. Clinical trials of ECT for the treatment of colorectal liver metastases^12,13^, hepatocellular carcinoma^14^, cholangiocellular carcinoma^15^, and pancreatic adenocarcinoma have been published^16^. Most of the included patients had advanced, unresectable tumours that were located in high-risk areas near large blood vessels and/or bile ducts where surgery or thermal ablation techniques were not feasible and/or safe^12–16^. Treatment response evaluation and follow up in these pilot trials were mostly performed using radiologic imaging^12–16^. Recently, we published the first detailed histopathological findings after ECT for colorectal liver metastases in a follow up paper^17^ to a phase I/II clinical trial^12^, where changes were described in treated metastases and normal tissue affected by electric fields 8–10 weeks after ECT treatment with bleomycin. Low amounts of residual tumour tissue were found in treated areas, with no viable tumour cells observed around larger vessels^17^. The functionality of blood vessels larger than 5 mm was mostly preserved; however, various levels of damage were observed in smaller blood vessels^17^.

Detailed data on the effects of ECT on normal liver tissue is lacking. Because of its nonthermal mechanism of action, deep-seated ECT is currently performed primarily for tumours near large blood vessels and other vital structures where no other treatment is possible due to the risk of serious complications^12,18^. Therefore, detailed knowledge of the extent and type of histological changes induced by ECT in normal liver tissue is crucial for further safe implementation of ECT into clinical practice. To determine whether ECT with bleomycin causes clinically significant damage to normal liver tissue with respect to large blood vessels and bile ducts, we designed a translational animal model study to investigate the effects of potentially risky ECT treatment strategies. An animal model study was selected due to the risks of possible vessel thrombosis, bleeding, and/or organ failure, as well as the need to obtain adequate tissue samples for histologic analysis. We chose pigs as our test animals, as porcine liver closely resembles human liver both anatomically and physiologically, thus electrode insertion inside and near large blood and bile ducts was possible^19^. Based on our previous findings in laboratory mouse studies and human clinical trials, we hypothesized that ECT with bleomycin will not cause clinically significant damage to normal liver tissue, and that no treatment effects on liver function and/or systemic inflammatory response will be observed^6,12,14,17^.

## Results

### Adverse events

Intraoperatively, no complications were detected related to electrode insertion, electric pulse delivery, or bleomycin administration. Despite not being able to synchronize electrical pulse delivery with the electrocardiogram (ECG), no heart rhythm abnormalities were recorded. Pigs showed no clinical signs of systemic inflammatory response or liver failure at any point during the study.

### Histological findings

**Figure 1:**
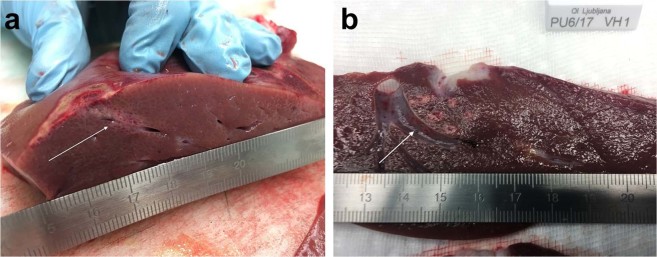
Gross examination of explanted livers. White arrows point to where variable linear (**a**) and fixed hexagonal (**b**) geometry electrodes were inserted and electric pulses were applied. All scales in cm.

Gross examination of explanted livers showed discernible areas where electric pulses had been applied, which lacked any macroscopically visible vessel thrombosis (Fig. 1a,b).

Histological analysis of tissue samples showed no difference between the use of ECT or electric pulses alone, demonstrating that bleomycin had no effect on liver parenchyma. Observed histological changes could be separated into two groups: acute/subacute changes seen two days after the procedure and chronic changes seen seven days after procedure.

#### Acute/subacute changes

**Figure 2:**
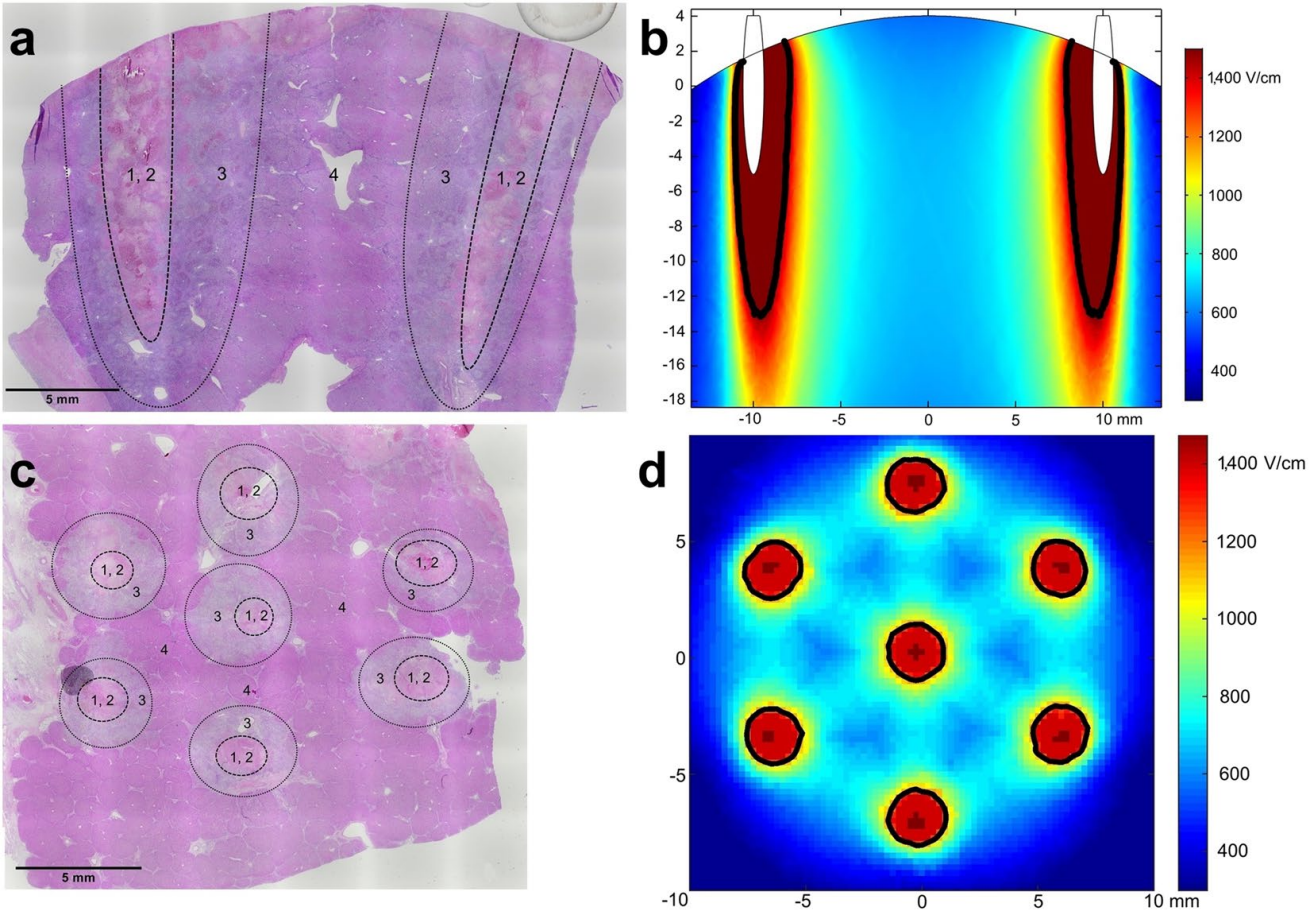
Representative images of the acute/subacute changes two days after the treatment. Zones 1 and 2 surround the electrode, Zone 3 represents partially damaged livery parenchyma, and Zone 4 represents intact liver parenchyma. The size of the zones depended on the type of the electrodes and number of pulses used. (**a**) Variable linear geometry electrodes (animal number 3; image shows the changes parallel to the insertion of the needles). (**b**) Numerical model showing the electric field distribution in the liver parenchyma between the two needle electrodes. Due to the angled cutting plane, the electrodes on image b are visible for only 5 mm. The thick black lines mark the threshold for irreversible electroporation. (**c)** Fixed hexagonal geometry electrodes (animal number 2; image shows the changes perpendicular to the insertion of the needles). (**d**) Numerical model showing the electric field distribution in the hexagonal needle array. The thick black lines mark the threshold for irreversible electroporation.

Acute/subacute changes were present in all animals that were euthanized two days after the procedure. Changes induced by the procedure were funnel-shaped and 5–8 mm wide. The diameter of changes was smaller when fixed hexagonal geometry electrodes were used. Clear zonation was present, and four zones of changes were recognized (Fig. 2).

**Figure 3:**
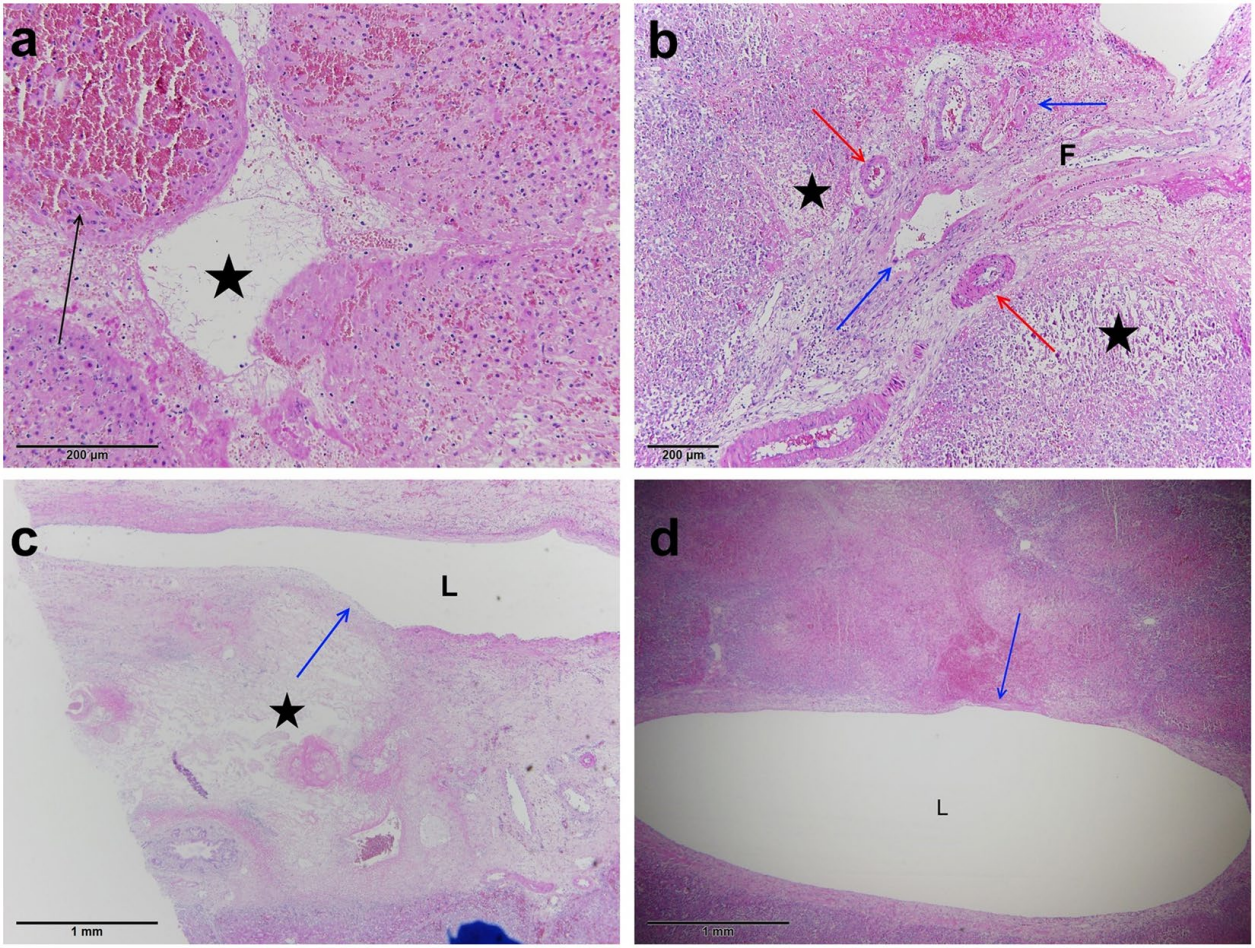
Close-up of the acute changes in Zones 1 and 2. (**a**) Central cavity caused by the insertion of the electrode (Zone 1, black star) filled with blood and coagulation necrosis (Zone 2, black arrow). (**b**) Damaged liver parenchyma (black stars) with ghost hepatocytes with pyknotic nuclei and partially damaged small venules (blue arrows) containing a fibrin thrombus (F). Arterioles are intact (red arrows). (**c**,**d**) Complete necrosis (black star) adjacent to the lumen (L) of a large hepatic vein. The vessel wall is partially damaged, with missing tunica muscularis (blue arrow), but no thrombosis is present.

Zones 1 and 2, surrounding the electrode: The central cavity caused by electrode insertion was filled with fibrin and blood (Zone 1) and surrounded by coagulation necrosis with complete loss of architecture (Zone 2) (Fig. 3a). Only ghost-hepatocytes were present with pyknotic nuclei, somewhat smudged chromatin, and shrunken, eosinophilic, and granular cytoplasm. Sinusoids were widened and were filled with erythrocytes, some neutrophils, and necrotic debris. The complete necrosis of the small blood vessels and bile ducts was present only in this zone (Fig. 3b). Where electrodes punctured the wall of the bigger vein, the architecture of the vessel wall was effaced with missing endothelium, but no thrombosis was present (Fig. 3c,d).

**Figure 4:**
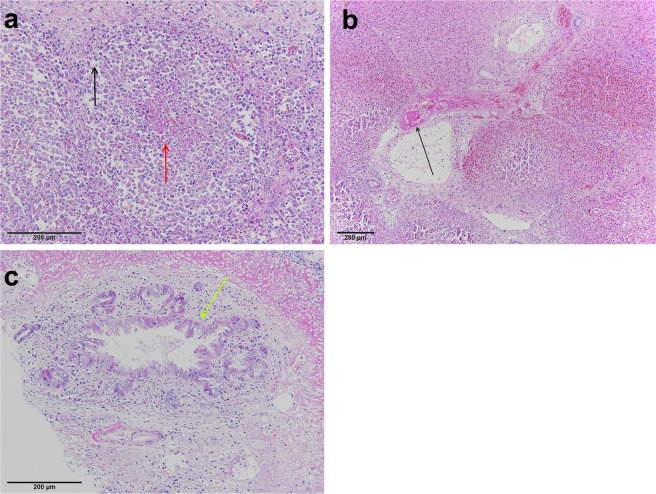
Close-up of the acute changes in Zone 3. (**a**) Damaged centri- and midlobular areas (red arrow) with the vital peripheral parts of the lobus (black arrow). (**b**) Fibrin thrombus (black arrow) in a damaged arteriole. (**c**) Undamaged bile duct epithelium (green arrow) near the coagulative necrosis.

Zone 3 of partially damaged liver parenchyma: In this zone, the damage was not distributed equally. It was most pronounced in the centri- and midlobular areas (Fig. 4a). Central veins were no longer visible, and the architecture of the central and middle parts of the lobules was destroyed. Sinusoids were distended and filled with blood and fibrin, with some hepatocytes showing changes similar to those in Zones 1 and 2 (pyknotic nuclei, smudged chromatin, and shrunken, eosinophilic, granular cytoplasm). Some neutrophilic exudate was present. Peripheral hepatocytes showed nuclei with open chromatin and shrunken cytoplasm, making the sinusoids appear wider, and many sinusoids were congested. Rough, dystrophic calcifications were already present focally. Changes in the blood vessels depended on their size, type (arterioles vs. venules), and distance from Zones 1 and 2. Vessels with a diameter less than 2 mm were incompletely affected, with a partial loss of the endothelial layer and partial necrosis or hyaline degeneration of the vessel wall. Changes were more pronounced in venules than in arterioles. Fibrin thrombi were present in the lumen of small damaged vessels (Fig. 4b). Vessels measuring more than 2 mm were intact. Epithelium of the bile ducts exhibited focal reactive atypia and increased mitotic activity; however, there were no signs of necrosis in the bile duct walls, irrespective of their size (Fig. 4c).

Zone 4 of preserved liver parenchyma: Regenerating, multinuclear hepatocytes were seen immediately surrounding Zone 3. Further away from the electrodes, approximately 3 mm when two variable linear geometry needle electrodes were used or approximately 2 mm when fixed hexagonal geometry needle electrodes were used, the liver parenchyma was intact.

#### Chronic changes

**Figure 5:**
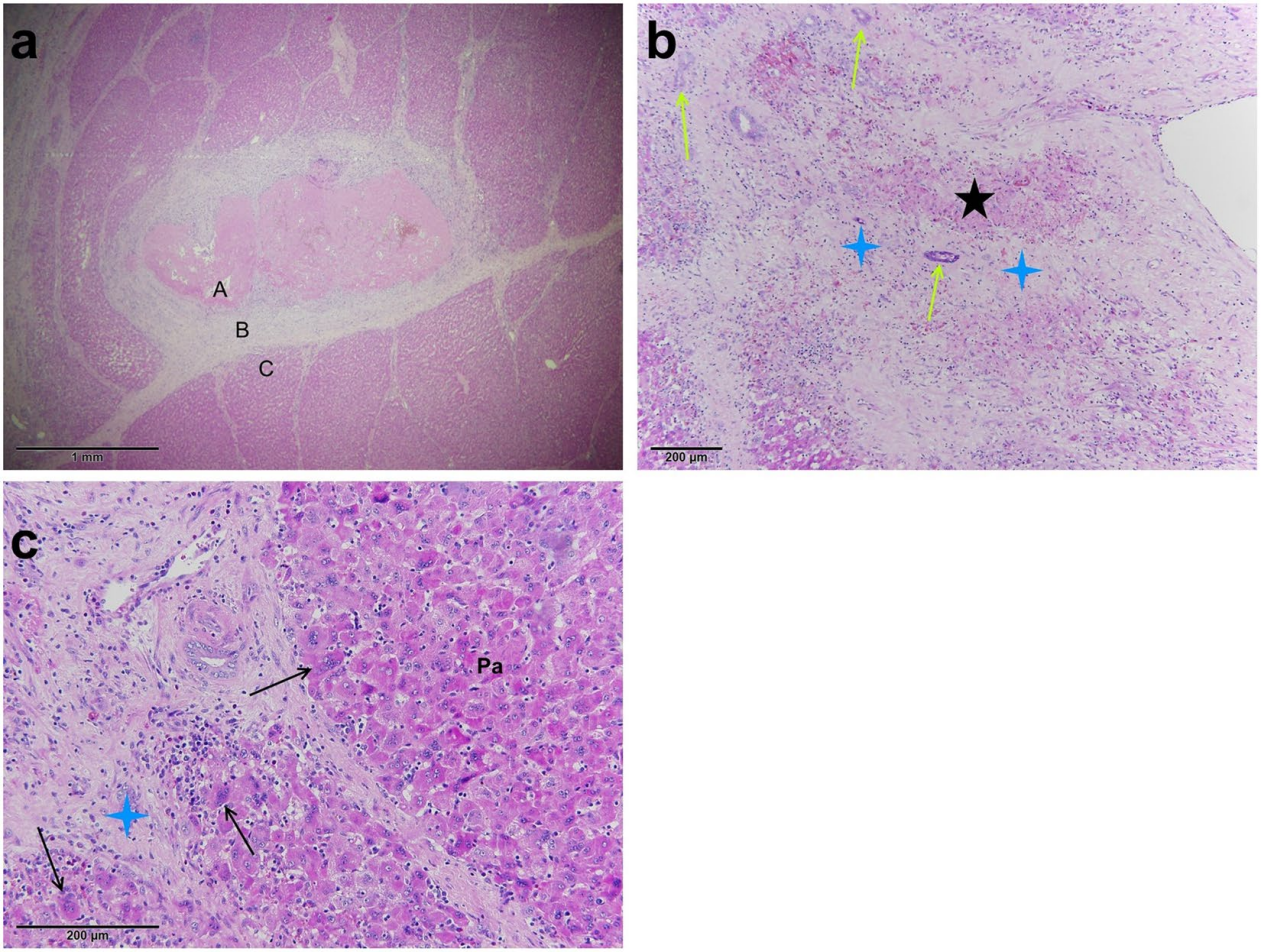
Representative image of the chronic changes seven days after treatment. (**a**) Three distinct zones were observed (Zone A of coagulation necrosis, Zone B of fibrotic proliferation, and Zone C of normal liver parenchyma with regenerating hepatocytes). (**b**) Residual necrosis (black star) with ingrowth of the granulation tissue in Zone A. Fibrotic changes (blue stars) with the proliferation of small bile ducts (green arrows) in Zone B. (**c**) Multinuclear regenerative hepatocytes (black arrows) were seen in Zone C on the margins of fibrotic changes (blue star), while the rest represent undamaged parenchyma (Pa).

Chronic changes were observed in animals that had been euthanized seven days after treatment. Chronic changes were narrower in comparison to the acute/subacute changes, measuring 4–5 mm at the largest diameter, and were primarily star-shaped. The hallmark of the chronic phase was fibrosis; however, zonation was observed again (Fig. 5a).

Zone A of coagulation necrosis and Zone B of fibrotic proliferation: Residual central defects with coagulation necrosis (Zone A) with the ingrowth of granulation tissue were seen. Peripheral parts of treated areas were nearly completely fibrotic (Zone B) with proliferation of the small bile ducts (Fig. 5b). Lymphocytes, plasma cells, eosinophils, and histocytes were present in fibrous tissue with focal foreign body reaction around rough calcifications and residual necrosis. Residual, partially damaged blood vessels of smaller diameter were observed in the fibrotic tissue. In two cases, probable foci of extramedullary haematopoiesis were noted.

Zone C of intact liver parenchyma with regenerating hepatocytes: Fibrotic areas surrounded by intact liver parenchyma had more regenerative marginal hepatocytes present than in the acute/subacute phase (Fig. 5c). The border between treated areas and residual vital liver parenchyma was not sharply delineated; some of the peripherally placed lobules were partially fibrotic and partially vital, reflecting the damage described in Zone 3 of acute/subacute changes.

### Laboratory test results

Laboratory analysis of arterial blood samples collected before treatment and before euthanasia showed a significant increase in the mean alanine aminotransferase (ALT) activity from 40.8 IU/L (s.d. = 9.7 IU/L) to 51.2 IU/L (s.d. = 13.9 IU/L) (Table 1). No statistically significant differences were observed for the other biomarkers of liver function and systemic inflammatory response.

**Table 1:** Experimental animal weights, treatment data and laboratory results.

Animal number	Weight	Time to euthanasia	Electrode type	Treatment	ALT activity (IU/L)^a^	
1	30 kg	2 days	Variable linear	ECT with bleomycin	57.6	*82.9*
2	32 kg	2 days	Fixed hexagonal	ECT with bleomycin	48.3	*56.1*
3	30 kg	2 days	Variable linear	Electric pulses	26.9	41
4^b^	33 kg	7 days	Variable linear	ECT with bleomycin	38.3	*49.6*
5^b^	28.7 kg	7 days	Variable linear	ECT with bleomycin	43.8	*45.3*
6^b^	32 kg	7 days	Variable linear	ECT with bleomycin	41	*44.1*
7	30 kg	7 days	Fixed hexagonal	ECT with bleomycin	30.5	*39.7*
8	30.5 kg	7 days	Variable linear	Electric pulses	39.7	*50.9*

Values in normal text are before treatment, and the values in italics are before euthanasia.

^a^Statistically significant difference, P = 0.005.

^b^We consider the use of two variable linear electrodes to be the riskiest ECT treatment strategy due to the high voltages used, therefore we repeated the experiment on three test animals.

## Discussion

The primary aim of our animal model study was to evaluate the histological changes in the liver induced by ECT with bleomycin. We found that ECT with bleomycin or the application of electric pulses alone caused well-defined changes around the inserted electrodes; however, no visible histological changes were observed in the liver parenchyma farther from the electrodes, despite the presence of an electric field. The most important finding of our study is that, despite the use of a potentially risky treatment strategy (that is, inserting at least one electrode into the lumen of large liver veins), no hemodynamically significant thrombosis was observed. We observed a variable level of small vessel damage (from partial damage with retained vessel patency to complete coagulation necrosis) in immediate proximity to the electrode insertion sites, with venules being the most affected, arterioles less so, and small bile ducts the least. More importantly, no significant damage or necrosis of larger blood vessels or bile ducts was seen anywhere in the parenchyma. We observed identical histological changes regardless of whether ECT or electric pulses alone were used; therefore, we can conclude that the use of bleomycin did not cause any additional damage to normal liver parenchyma. As our secondary study aim, we used standard laboratory blood tests to assess biomarkers of liver damage and inflammatory response. We found only slightly increased ALT activity, which is to be expected due to areas of coagulation necrosis of the liver parenchyma seen upon histologic examination, and no signs of systemic inflammatory response.

The observed histological changes could be due to several factors. One reason for histological changes could be temperature increases during electric pulse delivery. Similar zones of coagulation necrosis, sinusoidal congestion with relative preservation of microvasculature, apoptotic hepatocytes, and inflammatory cell infiltration have also been described after histological analysis of liver parenchyma treated with irreversible electroporation in pigs^20–22^. Irreversible electroporation uses electric pulses similar to those of ECT (albeit with a higher voltage-to-distance ratio and a higher number of pulses per electrode pair) without the use of cytotoxic drugs, to cause nonthermal cell death^23^. Large blood vessels and bile ducts seem to be largely unaffected by ECT, as well as irreversible electroporation, in which the collagen matrix scaffolding of vessels is supposedly preserved^24,25^. Electrode temperatures during treatment with irreversible electroporation have been shown to exceed 50 °C^23^, which is considered the threshold for thermal tissue damage^26–28^. There is currently no published data on electrode temperature during ECT, which, compared to irreversible electroporation, uses a lower voltage and significantly lower number of pulses and should, therefore, have less pronounced thermal tissue effects. However, three-dimensional finite-element analysis of joule heating for similar electric pulses (1,500 V/cm, 8 pulses, 100 μs, 1 Hz) and linear variable electrodes demonstrated that the temperature surrounding the electrode did not exceed 44 °C^29^. Therefore, we can presume that observed changes were not due to inadvertent thermal damage to the tissues caused by ECT.

Another possible mechanism that could cause the observed histological changes is irreversible cell electroporation of liver parenchyma around the electrodes due to a high local electric field strength^30–33^. This would explain why the diameter of changes was larger when we used variable linear geometry electrodes (1.2 mm diameter, 2,000 V applied between them), as opposed to fixed hexagonal geometry electrodes (0.7 mm diameter, 730 V applied between them). Based on numerical modelling and histological changes, the threshold for irreversible electroporation was 1,500 V/cm local electric field strength for linear geometry electrodes and 1,200 V/cm for hexagonal geometry electrodes (Fig. 2b,d). These values were higher than previously reported for the rabbit liver (700 V/cm)^33^, but similar to recently reported values for mouse tumours (1,800 V/cm)^34^. The higher threshold for irreversible electroporation with linear geometry electrodes is consistent with the lower number of pulses delivered (eight pulses, as opposed to 96 pulses delivered with hexagonal geometry electrodes), as the same voltage to distance ratio (1,000 V/cm) was used for both types of electrodes^35^. However, it is worth repeating that no clinically important changes were detected, as the observed histologic changes were limited to only a few millimetres of liver parenchyma around the electrodes, despite our treatment protocol being more aggressive than what is currently used for ECT in clinical practice.

ECT is mostly performed with palliative intent in current clinical practice^4,36^. Despite the proven clinical response of tumours to ECT, little is known about the effects of ECT in both tumour and normal tissue, as tissue specimens after treatment are rarely collected and analysed^37^. Therefore, only two clinical studies have reported histological findings after treatment with ECT, one for patients with colorectal liver metastases^12^ and one for patients with melanoma skin metastases^37^. In our previous study^12^, we reported coagulation necrosis in both tumour and normal liver tissue when ECT was used for the treatment of colorectal liver metastases, with variable levels of damage to blood vessels smaller than 5 mm and the preservation of larger blood vessels^17^. In this study, less damage to small blood vessels was observed. This could be because all patients included in our previous study^12^ were pre-treated with chemotherapy (in some cases combined with biological drugs or radiation therapy) prior to undergoing ECT, which could explain the greater sensitivity of liver parenchyma to additional damage^17^. Some of the included patients also had their right portal vein ligated at the time of ECT to enable a two-step liver resection^12^, which causes ischemia and possible thrombosis due to reduced blood flow^17^. Another possible explanation for the reduced damage to small blood vessels observed in the present study is the so-called vascular disrupting action of ECT, in which tumour endothelial cells are selectively destroyed with sparing of normal vasculature^6,38^. No significant bile duct damage was observed in our previous study^12,17^. In comparison, a study by Bigi *et al*. suggested apoptotic cell death as the primary mechanism of action when ECT was used to treat patients with melanoma skin metastases^37^. In our present study we performed experiments in an animal model with a normal liver that lacked tumour cells, thus we cannot speculate further about ECT tumour effects. Bigi *et al*. also observed few histological signs of vascular damage^37^, which was limited to endothelial cells, in line with our previous^5^ and current observations. In our present study, as well as in our previous study^12,17^ and in a study by Bigi *et al*.^37^, regenerative tissue changes with fibrosis formation and immune cell aggregation were observed, which are thought to be important for an additional immune system response to tumour antigens released after ECT.

We observed no clinical or biochemical signs of liver failure or systemic inflammation in test animals after the procedures. Although this study was not designed for testing of statistical significance and was therefore underpowered, a significant increase in ALT activity was found before euthanasia. Increased ALT activity was expected due to coagulation necrosis of small parts of the liver parenchyma. Transiently elevated activity of liver enzymes has also been described after irreversible electroporation in both pigs^39^ and humans^40^. A clinical study by Froud *et al*. described much higher peaks of ALT activity at postoperative day two than observed in our study; however, it is unclear whether this is due to the use of irreversible electroporation vs. ECT or because treatment was performed on human and not porcine liver^40^. Despite the dramatic rise in enzyme levels, no abnormal liver function was noted, and changes observed by Froud *et al*. were described as safe and self-limiting^40^. In our study, we found no significant increase in serum inflammatory markers after treatment. This can be contrasted to a marked increase in serum inflammatory markers found after the radiofrequency ablation or cryotherapy of porcine liver^41^.

**Figure 6:**
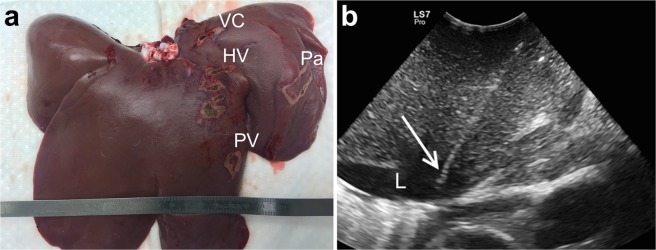
Electrode positioning. (**a**) Insertion sites of variable linear geometry needle electrodes are marked with electrocautery on an explanted pig liver (VC - caudal vena cava, HV - middle left hepatic vein, PV - left portal vein, Pa - peripheral liver parenchyma). (**b**) Intraoperative ultrasound image of one of the variable linear geometry needle electrodes (white arrow) inserted into the lumen (L) of the caudal vena cava during the treatment.

There are several limitations to our study. Following the principle of the 3Rs (replacement, reduction and refinement), we performed ECT on only a limited number of animals, which precluded more statistical analysis of the obtained data. We tried to overcome the limitation of having a small number of experimental animals by applying electric pulses in four different anatomical locations of the liver in each animal to collect more data (Fig. 6a). To further reduce the number of experimental animals, we decided against euthanasia shortly after the procedure to look for acute changes in liver blood flow, using radiologic methods instead. We expect to report these findings in a separate paper. As the liver already showed signs of regeneration and fibrosis after seven days, the development of further clinically important changes in the liver parenchyma or blood vessels is highly unlikely, and we do not consider the lack of a longer follow up a limitation of our study.

In conclusion, we found no hemodynamically significant thrombosis or other clinically important damage to the large liver blood vessels. Observed histological changes did not differ between ECT and electric pulse only treatments, demonstrating that bleomycin did not affect normal cells in liver. We observed no necrosis of the large bile ducts. No clinical or laboratory findings suggested impaired liver function or systemic inflammatory response after the procedure. No complications directly related to the ECT procedure were observed. After seven days, we already observed histological regenerative changes. Therefore, we can conclude that ECT with bleomycin does not cause clinically significant damage to normal liver tissue including large liver blood vessels and bile ducts, even when used with potentially risky treatment strategies. Our findings further support the use of ECT with bleomycin as a safe, efficient treatment for tumours near large blood vessels in the liver.

## Methods

### Experimental animals and study design

To evaluate the effects of ECT with bleomycin on liver histology, function, and systemic inflammatory response, an *in vivo* animal model study was conducted on eight female pigs (*sus scrofa domesticus*) that were hybrids of Landrace and Large White. Pigs were procured from an authorized swine breeder 3–17 days before the procedure. They were housed in separate indoor straw-bedded pens sized 1.25 × 4.40 m, enabling visual and audible contact. They were exposed to a natural light/dark cycle and kept at an ambient temperature of 20–23 °C with relative air humidity of 50–65%. They were fed twice daily with commercial feed for grower pigs and had unlimited access to tap water from nipple waterers. A caretaker monitored the condition of the animals three times daily, with continuous monitoring for the first 24 h after treatment.

ECT with bleomycin was performed on six pigs, while two pigs received electric pulses alone to serve as a control group to rule out possible bleomycin cytotoxic effects and thermal damage identification (Table 1). Electric pulses were delivered in four anatomic locations in the liver of each pig using both variable linear and fixed hexagonal geometry needle electrodes (Fig. 6a). The study endpoint was euthanasia on postoperative day two or seven for liver explantation and the collection of tissue specimens and blood samples. The number of experimental animals, the blood sample collection, and the euthanasia times were comparable to previously published studies of ablation techniques in porcine liver^41–43^. The study design, regarding bleomycin dosage and electric pulse parameters, was based on the ESOPE study for cutaneous tumours^44^ and the ECT for Colorectal Liver Metastases study^12^.

Regulatory approval for this study was obtained from the National Ethics Committee at The Administration of the Republic of Slovenia for Food Safety, Veterinary, and Plant Protection (Approval number: U34401-1/2017/4, Approval date: 17.03.2017). Experimental animals were reared according to the European Council directive for minimum standards for the protection of pigs (2008/120/EC). All procedures complied with relevant national and European legislation (2010/63/EU).

### Treatment protocol

Pigs were sedated with 10 mg/kg intramuscular (im) ketamine (Bioketan, Vetoquinol, Lure Cedex, France), 0.5 mg/kg im midazolam (Midazolam Torrex, Torrex Chiesi Pharma GmbH, Wien, Austria), and 0.5 mg/kg im butorphanol (Butomidor, Richter Pharma AG, Wels, Austria). Animals were weighed using measuring tape for pigs (Rondo combi, Kruuse, Langeskov, Denmark). Their body surface area was estimated using the formula: 100 × body surface area (m^2^) = 7.98 × body weight^2/3^ (kg)^45^. Intravenous access was obtained, and anaesthesia was induced with 1–2 mg/kg iv propofol (Norofol, Norbrook Laboratories, Newry, Northern Ireland). After endotracheal intubation, anaesthesia was maintained with a mixture of isoflurane (Isoflurin, Vetpharma Animal Health, Barcelona, Spain) and oxygen, 4 µg/kg/h iv fentanyl (Fentanyl Torrex, Chiesi, Parma, Italy), and 0.5 mg/kg iv rocuronium (Esmeron, NV Organon, Oss, Netherlands). Intermittent positive pressure ventilation was used (Ventilog, Dräger Tiberius 800, Lübeck, Germany). Additionally, 4 mg/kg iv carprofen (Rimadyl, Zoetis Belgium SA, Louvain-La-Neuve, Belgium), 9 mg/kg im amoxicillin with clavulanic acid (Klavuxil, Genera, Rakov potok, Croatia), and 10 ml/kg/h iv saline (NaCl 0.9%, B Braun, Melsungen, Germany) were administered. Blood pressure was monitored noninvasively using an ultrasonic Doppler flow monitor (Model 811, Parks Medical Electronics, Beaverton, OR, USA). ECG, end-tidal carbon dioxide tension, and arterial oxygen saturation were monitored using a multiparametric monitor (BLT M9000 VET, Guangdong Biolight Meditech, Zhuhai, China).

Anesthetized pigs were secured to the operating table in a supine position. The hair on the abdomen was clipped, and the surgical field was prepared aseptically. A median laparotomy was performed, and the liver was mobilized. Intraoperative ultrasound guidance (Logiq S7 Pro, GE, Milwaukee, WI, UA or M9, Mindray, Shenzhen, China) was used for the insertion of either two variable linear or seven fixed hexagonal geometry needle electrodes (IGEA SpA, Carpi, Italy) into the lumen of the caudal vena cava and adjacent liver parenchyma (Fig. 6b). The active parts of the two variable linear geometry electrodes were 3 cm long and had a diameter of 1.2 mm each. They were placed in a custom-made plastic holder to keep them parallel and exactly 2 cm apart. The single-use fixed hexagonal geometry electrodes consisted of a round plastic holder with seven hexagonally placed needle electrodes 0.7 mm each in diameter, which were spaced 0.73 cm apart.

Animals in the ECT experimental group received 15,000 IU/m^2^ iv bleomycin (Bleomycinum, Heinrich Mack. Nachf. GmbH, Illertissen, Germany). Between eight and 28 minutes after bleomycin bolus administration, eight electric pulses (variable linear geometry electrodes) or 96 electric pulses (fixed hexagonal geometry electrodes) were delivered using the electric pulse generator (Cliniporator, IGEA SpA). Each pulse lasted 100 µs. The voltage between electrode pairs was set to either 2,000 V (variable linear geometry electrodes) or 730 V (fixed hexagonal geometry electrodes), corresponding to a 1,000 V/cm voltage to distance ratio. The frequency was 1 Hz in the case of the variable linear geometry electrodes and 5 kHz for the fixed hexagonal geometry electrodes. The electric field in the treated area was computed with the finite element method using the software package Comsol Multiphysics (COMSOL AB, Stockholm, Sweden) with MATLAB (Mathworks, Natick, MA, USA)^26^. The delivery of electric pulses was not synchronized with ECG, as the resting heart rate of the pigs was above the electric pulse generator cut-off rate.

After pulse delivery, electrodes were removed from the liver parenchyma, and treated areas were marked with electrocautery to help with identification during histological examination. Care was taken not to use electrocautery directly on electrode insertion sites. Haemostasis of the liver was obtained by warm gauze packing. The same procedure of electrode insertion with electric pulse delivery was then repeated for one of the portal veins and one of the hepatic veins. Therefore, the hepatic arteries and the common liver bile duct were also encompassed within the treated area. The fourth electrode insertion location was randomly selected in the parenchyma of the left lateral liver lobe, to serve as a control for liver parenchyma changes away from large blood vessels. The two animals in the electric-pulse-only control group received no bleomycin; however, the rest of the procedure protocol was identical to the experimental ECT group.

Muscle fascia and skin were closed with absorbable sutures. Wound edges were infiltrated with L-bupivacaine (Chirocaine 0.5%, Abb Vie, Campoverde, Italy) with a dose not exceeding 2 mg/kg. Anaesthesia was discontinued, and muscle relaxation was reversed using 0.05 mg/kg iv neostigmine (Neostigmin, Rotexmedica, Trittau, Germany) with 0.03 mg/kg iv atropine (Atropinum sulfuricum Nycomed, Takeda Austria, Linz, Austria). A single dose of 0.15 mg/kg im methadone (Synthadon, Produlab Pharma, Oudewater, Netherlands), 75 µg/h fentanyl transdermal patch (Durogesic, Janssen Pharmaceutica, Beerse, Belgium), and 4 mg/kg/24 h po carprofen (Rimadyl, Zoetis Belgium SA) were used for postoperative analgesia. To prevent wound infection, 9 mg/kg/24 h po amoxicillin with clavulanic acid (Clavaseptin, Vetoquinol) was given continuously. Pigs had free access to water and food after surgery. On postoperative day two or seven, pigs were reanaesthetized using the same protocol. They were subsequently euthanized using 3 ml/10 kg iv T61 euthanasia solution (Intervet, Boxmeer, Netherlands). After confirmation of cardiac arrest using a Doppler flow monitor, the liver was explanted for histological analysis.

### Experimental outcomes

#### Histological examination

Tissue specimen collection was accomplished within one h of liver explantation. The liver surface was inspected and imaged (Fig. 6a). The liver was then cut into 5 mm slices, which were macroscopically examined, and the changes observed in treated areas were imaged. Tissue samples were collected from both macroscopically altered and normal liver parenchyma. Samples were fixed in 10% buffered formaldehyde for 24 h. From paraffin block samples, 2–3 µm thick sections were cut and stained with H&E. Microscopic examination of specimens was performed, and all histological changes and characteristics of liver tissue were noted and imaged.

#### Laboratory analysis

Before treatment and again before euthanasia, blood samples were placed into collection tubes (BD Microtainer and BD Vacutainer, Becton Dickinson, Franklin Lakes, NJ, USA) to determine complete blood count, prothrombin time, and biochemistry profile. Samples for complete blood count were analysed immediately (Advia 120, Siemens, Munich, Germany). Sodium citrate blood tubes with samples for determination of prothrombin time were centrifuged at room temperature for 15 minutes at 2,000 g followed by analysis with a coagulometer (H. Amelung KC 1A micro, H. Amelung, Lemgo, Germany). Blood samples for the determination of serum concentration of creatinine, urea, albumin, bile acids, and ALT activity were left still for 30 minutes to clot at room temperature, followed by centrifugation at 1,300 g for 10 minutes at 4 °C and then analysis (RX Daytona, RANDOX, Crumlin, UK). Blood samples for the determination of amylase activity and C-reactive protein concentration were left still for at least 30 minutes to clot and then centrifuged at 3,000 rpm for 10 minutes at room temperature. Afterwards, the serum was transferred to Eppendorf tubes (Golias Labortehnika, Ljubljana, Slovenia) and stored at −20 °C until analysis for amylase activity (Cobas 8000 Modular Analyzer, Roche Diagnostics, Basel, Switzerland) and C-reactive protein (Pig CRP ELISA kit, Abcam, Cambridge, UK) serum concentration. Blood samples for the determination of glucose and lactate concentrations were collected into lithium-heparinized syringes (Gaslyte arterial blood sampler, Vital Signs, Inc., Englewood, CO, USA) and analysed immediately (Rapid point 500, Siemens Healthcare, Erlangen, Germany).

### Statistical analysis

Our study was designed to report primarily descriptive results; therefore, no valid statistical comparisons were possible between groups. Preoperative and pre-euthanasia laboratory values were compared using paired-sample t-tests (IBM SPSS Statistics version 20.0, IBM, Armonk, NY, USA). A P value of less than 0.05 was considered statistically significant.

## Data Availability

The datasets generated during and/or analysed during the current study are available from the corresponding author on reasonable request.
